# P035. Headache prevalence and disability among Italian adolescents aged 11-15 years: a population cross-sectional study

**DOI:** 10.1186/1129-2377-16-S1-A148

**Published:** 2015-09-28

**Authors:** Erika Maffioletti, Federica Ferro, Ennio Pucci, Marco Giovanni Persico, Silvia Molteni, Giuseppe Nappi, Umberto Balottin

**Affiliations:** Child Neuropsychiatry Unit, IRCCS National Neurological Institute “C. Mondino”, Pavia, Italy; University Centre for Adaptive Disorders and Headache (UCADH), Unit of Pavia I, IRCCS “C. Mondino” Foundation, University of Pavia, Pavia, Italy; Nuclear Medicine Unit, IRCCS San Matteo Hospital Foundation, Pavia, Italy

## Background

Headache is a very common health problem among adolescents, causing school and social disability [1]. This is the first Italian cross-sectional school-based study to use the new ICDH-3 beta classification system. The aim was to determinate the prevalence of headache and to investigate headache-related disability in a school-population aged 11-15.

## Methods

Three hundred and seventy-six adolescents aged 11-15 years, living in the Pavia province (Italy), were recruited for this cross-sectional school-based study. They were assessed about their headache using a medical history questionnaire; headache diagnosis was made according to the new classification system of the International Headache Society (ICDH-3 Beta). Headache-related disability was assessed by means of PedMIDAS (Pediatric Migraine Disability Assessment Score).

## Results

Of the 376 students enrolled 91 (24.2%) had headache: 60.44% girls (n=55) and 39.56% boys (n=36). At the first diagnosis, the prevalence of any migraine was 28.6% (n=26), any tension-type headache (TTH) 60.4% (n=55), any medication-overuse headache 3.3% (n=3) and “unclassifiable” headache was 7.7% (n=7) (Table 1). All types of chronic daily headache (CDH) and probable chronic daily headache (PCDH) were 23.07% (n=21) and 14.3% (n=13) of participants suffering from headache fulfilled criteria for more than one headache diagnosis (Table 2). No significant difference was found in PedMIDAS score between adolescents with migraine and those with TTH (p = 0.5074), neither between girls and boys (p = 0.961). More than half (53.84%, n=49) of headache types had a low PedMIDAS score, corresponding to no or low disability (Grade I), 25.27% (n=23) of adolescents had mild disability (Grade II), 6.6% (n=6) had moderate disability (Grade III) and 14.28% (n=13) had a severe disability (Grade IV) (Table 3). PedMIDAS score was highest in adolescents with chronic migraine (range from 13 to 176), but severe disability (Grade IV) was the same in chronic migraine (50%, 2 students of 4) and chronic TTH (50%, 7 students of 14). Of the 91 adolescents, only 4 (4.4%) were referred to the Headache Center of the Neuropsychiatric Clinic of Pavia, 1 was referred to an oculist and 5 were followed by a general practitioner, the others did not refer to anyone.

**Table 1:** Prevalence of ICDH-3 Beta headache disorders for the total sample and by sex.

	Total number of adolescents with headache (first diagnosis)	Girls	Boys
**Any headache**	91 (24.2% of 376)	55 (60.44%)	36 (39.56%)
**Migraine without aura**	10 (11% of 91)	2 (3.6% of 55)	8 (22.2% of 36)
**Migraine with aura**	2 (2.2%)	1 (1.8%)	1 (2.7%)
**Probable migraine without aura**	7 (7.7%)	5 (9%)	2 (5.5%)
**Probable migraine with aura**	3 (3.3%)	1 (1.8%)	2 (5.5%)
**Chronic migraine**	3 (3.3%)	3 (5.4%)	0
**Probable chronic migraine**	1 (1.1%)	1 (1.8%)	0
**Infrequent TTH**	4 (4.4%)	1 (1.8%)	3 (8.3%)
**Frequent TTH**	29 (31.8%)	22 (40%)	7 (19.4%)
**Probable infrequent TTH**	3 (3.3%)	2 (3.6%)	1 (2.7%)
**Probable frequent TTH**	5 (5.5%)	3 (5.4%)	2 (5.5%)
**Chronic TTH**	10 (11%)	7 (12.7%)	3 (8.3%)
**Probable chronic TTH**	4 (4.4%)	1 (1.8%)	3 (8.3%)
**Medication-overuse headache**	3 (3.3%)	2 (3.6%)	1 (2.7%)
**Unclassifiable**	7 (7.7%)	4 (7.3%)	3 (8.3%)

**Table 2:** Prevalence of ICDH-3 Beta headache disorders for the total sample: other types of headache.

	Total number of adolescents with more than one type of headache	First Diagnosis
**Migraine without aura**	2	Chronic TTH (n=1) Frequent TTH (n=1)
**Migraine with aura**	8	Chronic TTH (n=2) Frequent TTH (n=5) Migraine with aura (n=1)
**Probable migraine with aura**	2	Chronic TTH (n=1) Frequent TTH (n=1)
**Medication-overuse headache**	1	Chronic Migraine (n=1)
**TOTAL**	13 (14.3% of 91)	Chronic TTH (n=4) Frequent TTH (n=7) Migraine with aura (n=1), Chronic Migraine (n=1)

**Table 3:** Headache-related disability (PedMIDAS Score) by diagnosis.

	Range	Grade I (0-10)	Grade II (11-30)	Grade III (31-50)	Grade IV (>51)
**Any Headache (n=91)**	0-176	49	23	6	13
**Migraine without aura**	0-33	6	3	1	0
**Miraine with aura**	6-8	2	0	0	0
**Probable migraine without aura**	2-18	5	2	0	0
**Probable migraine with aura**	4-12	2	1	0	0
**Chronic Migraine**	13-176	0	1	0	2
**Probable Chronic Migraine**	7	1	0	0	0
**Infrequent TTH**	1-5	4	0	0	0
**Frequent TTH**	0-69	15	12	0	2
**Probable infrequent TTH**	0-1	3	0	0	0
**Probable frequent TTH**	3-33	3	1	1	0
**Chronic TTH**	5-120	1	1	4	4
**Probable Chronic TTH**	15-130	0	1	0	3
**Medication-overuse Headache**	13-94	0	1	0	2
**Unclassifiable**	0-5	7	0	0	0

## Conclusions

Headache is common in adolescents and can affect schoolwork and social activity. Thus, it is important to raise the awareness among general practitioners, families and teachers, so that they can identify headache in adolescents in its early stages and refer them for appropriate treatment.

Written informed consent to publish was obtained from the patient(s).

## Conflict of interest

None declared.

## References

[1] Krogh AB, Larsson B, Linde M (2015). Prevalence and disability of headache among Norwegian adolescents: a cross-sectional school-based study. Cephalalgia.

